# The New York Institute of Stomatology

**Published:** 1904-02

**Authors:** 

**Affiliations:** The New York Institute of Stomatology


### Reports of Society Meetings.

### THE NEW YORK INSTITUTE OF STOMATOLOGY.

A MEETING of the Institute was held at the "Chelsea," No. 222 West Twenty-third Street, New York, on Friday, November 6, 1903, the President, Dr. J. Morgan Howe, in the chair.

The minutes of the last meeting were read and approved.

Dr. Wooley, as chairman of the committee, presented a memorial to the late member of the Institute, Dr. Louis Shaw. The memorial was adopted and ordered recorded in the proceedings of the society. The secretary was instructed to send a copy of the same to the family of the deceased.

Owing to the importance of the paper of the evening, the communications on theory and practice were omitted.

Dr. D. B. Kyle, of Philadelphia, read a paper entitled "The Relation of the Chemistry of the Saliva (Sialo-Semeiology) and Nasal Secretions to Diseases of the Mucous Membrane of the Mouth and Upper Respiratory Tract."

(For Dr. Kyle's paper, see page 94.)

Dr. Kirk wished first to express his thanks to the Institute for the privilege of being present and listening to such an instructive and interesting paper. His own interest in the subject had extended back about three years, and his researches and study had been confined principally to one aspect of the subject only,—the hyperacid diathesis. He had learned a great deal to-night about the chemical side of the hypoacid diathesis. Dr. Kirk was pleased to confirm, from his own observation, the chemical aspect of the problem, so far as the essayist had stated it.

Speaking of the susceptibility to necrotic conditions in hypoacid cases, Dr. Kirk asked if the essayist meant to imply that the necrosis was chemical and not bacterial. He thought it generally conceded that the hypoacid case was the one most susceptible to bacterial infection. He did not think the essayist intended to convey the idea that necrotic conditions were produced by the hypoacid condition, but that diathetic hypoacidity was a condition that made these cases susceptible to bacterial infection, which infection caused the necrosis. Dr. Kirk thought there was a direct relationship between the food habit and the diathetic state. It had been recently shown that in Oriental countries, where the diet was chiefly vegetable, while caries of the teeth was not prominent, pyorrhœa alveolaris existed to a tremendous degree. A starchy diet evidently tended in certain cases to the production of a hypoacid condition.

Regarding the exact interpretation of the microscopic appearances of crystallized saliva deposits, Dr. Kirk had for some time been endeavoring to determine, one at a time, the chemical nature of some of the substances that we see. The essayist had developed the thought that a high ammonium content in saliva indicated a hypoacid diathesis. From his own point of view he had settled upon large quantities of the so-called neutral sodium phosphate rather than the ammonium as indicative of the hypoacid diathesis. Sodium phosphate is an extremely interesting compound. The early writers on the chemistry of the saliva and the urine had gone astray in their interpretation of its appearance under the microscope. Chemical investigation in this field by the ordinary methods of the laboratory is extremely difficult. In many respects it is beyond the reach of such methods. When Michaels suggested the use of the polariscope he gave us a means in advance of anything that had been accomplished. The neutral sodium phosphate occurring so abundantly in the hypoacid conditions had been put down in earlier works as ammonium chloride. The appearance of the two compounds is very similar until a special study has been made of them. However, the so-called neutral sodium phosphate is really alkaline, and the alkalinity of the normal saliva may be measured in terms of sodium phosphate, with sodium carbonate and bicarbonate. Both are found in normal and in hypoacid saliva, but in different amounts.

When we came to the hyperacid individual we had an entirely different aspect. Here we had an individual producing large quantities of $CO_2$, and more rapidly than it can be eliminated, the excess being dammed up in the plasma of the blood, existing as $H_2CO_3$. The normal reaction by which this over-acidity is taken care of by the renal epithelium is $H_2CO_3 + HNa_2PO_4 = H_2NaPO_4 + HNaCO_3$, but when the conditions are such as to produce larger quantities of carbonic acid than the kidneys can eliminate as sodium acid phosphate, other epiblastic structures take on the same action and there is a higher acidity of the saliva as well as the urine. A curious chemical feature of this matter was that the acid sodium phosphate and the neutral sodium phosphate might exist in the same solution without neutralizing each other, giving a solution that would turn blue litmus-paper red and red litmus-paper blue, the so-called amphoteric reaction. Thus we sometimes find saliva that would give both an acid and an alkaline reaction.

Certain conditions of the teeth exist in these hyperacid conditions, in the shape of erosions of the teeth. The teeth themselves were also of a peculiar character, becoming over-calcified and hard, translucent and vitreous in texture.

The appearance of the lactophosphate of calcium in the saliva and urine indicates certain diseases of the liver. Dr. Kirk had been led to this conclusion from reading the researches of Minkowski, who had found, in a series of experiments upon geese, where he had removed the liver, that they then excreted, instead of uric acid and the urates, the ammonium lactate, and he deduced from these experiments that one of the functions of the liver was to convert ammonium lactate into urates; so the appearance of lactates in excessive amounts in the excretions, especially in the urine, is indicative of a disordered liver function.

Dr. Kirk also called attention to the coincidence of neurasthenic states with the appearance of oxalic acid in combination with sodium, both in the saliva and in the urine. He had determined to his own satisfaction that the presence of these oxalates of the alkali bases in the secretions was metabolic in origin, and that the oxalic acid was not derived from the ingested food, for he found them to persist in patients from whom all oxalate-carrying food-substances were excluded. The oxaluria of sodium oxalate was only possible of determination by the polariscope and a stage of the disorder considerably antedating the period when calcium oxalate makes its appearance in the urine and saliva. The nervous phenomena coincident with the sodium oxalate oxaluria are constant and pronounced.

In conclusion Dr. Kirk stated that he was doing all he could in this direction, and that he was glad to learn that Dr. Kyle was also interested and had taken up the work along the same line.

The President asked whether there was any test, readily applied, by which the hyperacid, the hypoacid, or the normal condition of the saliva could be ascertained.

Dr. Bogue thought that if Dr. Kirk would consent to tell us the methods by which the various oxalic acid compounds could be detached by means of the polariscope, a partial answer would be given to the President's question.

Dr. Kirk stated that in the saliva of the hypoacid cases and in the saliva of the normal individual he had never found the oxalates, acid phosphates, and urates. In the hypoacid individual there was a preponderance of the neutral sodium phosphate, with ammonium salts and chlorides, and the individual himself was of a different type from the hyperacid, in whom there was an absence of caries and a tendency to erosion.

He could not very well describe the polariscope. It so happened that many of the waste products of the urine and saliva had the property of rotating the plane of polarized light. Hence these waste products of abnormal nutrition were readily recognized by the polariscope. The abnormal ones were the most readily polarizable. Under the microscope without the polariscope there was very little to distinguish them.

Dr. Kyle, in closing the discussion, stated, in regard to the relation of the hypoacid state to necrosis, that the strongly alkaline condition of the saliva was more irritating to the mucous membrane than the hyperacid condition. This irritation would make the tissue more susceptible to bacterial infection and beginning necrosis. Of course, necrosis does occur without bacterial infection. What he meant, however, was that this alkaline condition was a strong predisposing factor.

He was pleased to hear from Dr. Milliken's report a confirmation of his theories. He was glad also to see from Dr. Kirk's statements that, although not working together, their results were practically the same.

He was extremely interested in the statements of Dr. Kirk relative to the determining of lesions of certain organs by the chemic constituents of the saliva. He believed this would be a very valuable means, in time, of determining the position and character of organic lesions.

Dr. Kyle closed by thanking the Institute for his kindly reception.

Dr. F. Milton Smith did not feel competent to discuss the paper, but he wished to express his sincere gratitude to the gentlemen who had taken part. He thought he could appreciate the embarrassment under which such men worked, and the fact that so many times they worked long and diligently with little apparent reward other than the feeling in their own hearts that they were doing the right thing and were bringing things to pass, although they might not seem to be appreciated. For this reason he wanted to express his great appreciation for the papers that had been read and the words that had been spoken. He would also relate a little experience that might give one of the gentlemen present a feeling of satisfaction.

Some ten years ago he had had the pleasure of hearing Dr. Kirk read a paper upon a subject which at that time seemed somewhat foreign to dental matters. The subject was "Infantile

Scorbutus." At the time he did not expect to meet with any such case in his practice, but within six months a lady came to him with a little one thirteen months old with a condition that he was able, from Dr. Kirk's description, to diagnose as infantile scorbutus. The child had been treated by various physicians for dental paralysis and rheumatism, but none had been able to recognize the true cause of the trouble. Following the suggestions of Dr. Kirk's paper, Dr. Smith suggested a course of treatment, and in six weeks' time the child was practically well. She had shortly been in his chair, and while there her mother had asked if she remembered Dr. Smith. The child had said that she did not. The mother said, "Dr. Smith saved your life." Dr. Smith stated that if any one saved the life of that child it was Dr. Kirk, for she certainly would have died had she not received proper treatment, and that speedily. He also said that we really did appreciate their work even if we did not discuss it as we would like to do.

Dr. C. F. Allan stated that it had been a great favor to the society to have this paper presented. It had been a great favor for Dr. Milliken to come so far in order to present his valuable clinical evidence, and it had also been a great favor to the society for Dr. Kirk to come here to-night and give us this information about the investigations he was making. Dr. Allan moved that the thanks of the Institute be extended to these gentlemen.

Motion carried unanimously.

Adjourned.

FRED. L. BOGUE, M.D., D.D.S.,
*Editor The New York Institute of Stomatology.*

